# TiO_2_ Nanoparticles Loaded with *Polygonum cuspidatum* Extract for Wound Healing Applications: Exploring Their Hemolytic, Antioxidant, Cytotoxic, and Antimicrobial Properties

**DOI:** 10.3390/nano15120926

**Published:** 2025-06-14

**Authors:** Gabriela Fletes-Vargas, Rogelio Rodríguez-Rodríguez, Natalha Vicentina Pinto, Kelly Cristina Kato, Guilherme Carneiro, Ana Paula Rodrigues, Helen Rodrigues-Martins, Hugo Espinosa-Andrews

**Affiliations:** 1 Departamento de Ciencias Naturales y Exactas, Centro Universitario de los Valles (CUVALLES), Universidad de Guadalajara, Ameca 46600, Mexico; 2 Department of Pharmacy, Faculty of Health and Biological Sciences, Universidade Federal dos Vales do Jequitinhonha e Mucuri—UFVJM, Campus JK, Rodovia MGT 367-km 583, n° 5000, Alto da Jacuba, Diamantina 39100-000, MG, Brazil; natalha.vicentina@ufvjm.edu.br (N.V.P.); kelly.kato@ufvjm.edu.br (K.C.K.); guilherme.carneiro@ufvjm.edu.br (G.C.); anapaula.rodrigues@ufvjm.edu.br (A.P.R.); helen.martins@ufvjm.edu.br (H.R.-M.); 3 Unidad de Tecnología Alimentaria, Centro de Investigación y Asistencia en Tecnología y Diseño del Estado de Jalisco, A.C., Camino Arenero # 1227, Col. El Bajío del Arenal, Zapopan 45019, Mexico; hespinosa@ciatej.mx

**Keywords:** polyphenols, antimicrobial, antioxidant, titanium dioxide, wound healing

## Abstract

The dry roots of *Polygonum cuspidatum* contain resveratrol, a compound known for its antimicrobial and protective effects against oxidative stress, which is associated with impaired wound healing. In this study, titanium dioxide nanoparticles (TiO_2_NPs) were loaded with a *P. cuspidatum* extract (TiO_2_-loaded extract NPs), and the resveratrol release profile, hemocompatibility, antioxidant, cytotoxic, and antimicrobial activities were evaluated. The results demonstrated that TiO_2_-loaded extract NPs exhibited antioxidant activity for DPPH (Inhibitory Concentration 50 (IC_50_) = 62.31 mg Trolox Equivalent (TE)/mL) and ABTS^+^ (IC_50_ = 4.8 mg TE/mL) assays, along with suitable hemocompatibility (3.02% at 10 mg/mL), in comparison with bulk TiO_2_ NPs. Additionally, temperature influenced the resveratrol release over time. The *P. cuspidatum* extract alone showed strong antibacterial activity, with a Minimal Inhibitory Concentration (MIC) of 5 µg/mL, TiO_2_-loaded extract NPs showed MIC values about 50 mg/mL, while bulk TiO_2_ NPs exhibited no antibacterial effect against the tested strains. In contrast, the *P. cuspidatum* extract, the TiO_2_-loaded extract NPs, and the bulk TiO_2_ NPs did not demonstrate antifungal activity against *Candida albicans* and *C. glabrata*. Moreover, TiO_2_-loaded extract NPs showed no cytotoxicity against the L-929 cell line at concentrations ranging from 1.5 to 150 µg/mL, unlike TiO_2_ NPs, which exhibited high cytotoxic concentrations between 9.4 and 300 µg/mL. These findings suggest that TiO_2_-loaded extract NPs effectively control the release of resveratrol and hold promises for applications in skin management and wound healing.

## 1. Introduction

Resveratrol is a polyphenol with various biological activities, including antioxidant, anticancer, anti-inflammatory, proliferative, and angiogenic properties. Among these, its antioxidant activity is considered the most relevant to its health-promoting benefits [1]. Resveratrol protects cells through its free radical scavenging properties, enabling it to modulate oxidative stress, which is commonly associated with chronic diseases and dysfunctional wound healing [2]. Wound healing is a complex and self-regulated biological process that restores tissue integrity [3]. However, the accumulation of reactive oxygen species (ROS), which induce oxidative stress, can disrupt the healing process and lead to cell death, inflammation, infection, necrosis, and scarring [4].

*Polygonum cuspidatum* Sieb. et Zucc, commonly used in traditional Chinese medicine, contains bioactive compounds such as resveratrol, piceid, and polydatin, as well as the anthraquinone emodin, mainly found in the roots [5]. Extracts from *P. cuspidatum* roots have been extensively studied for their biological activities, including anti-inflammatory, analgesic, antioxidant, antimicrobial, anticancer, and wound healing effects [6]. In this context, Wu and Huang [7] evaluated the wound-healing effect of *P. cuspidatum* extract in an animal model. The authors reported that *P. cuspidatum* extract promotes re-epithelialization and angiogenesis by activating TGF-β1, which induces myofibroblast proliferation. Similarly, Hadzik et al. [8] reported that a decoction of *P. cuspidatum* rhizome stimulates gingival fibroblast proliferation and increases collagen type III synthesis, contributing to oral wound healing. Additionally, Zhou et al. [9] demonstrated that resveratrol accelerates skin wound healing by regulating Nrf2 and Mn-SOD expression, which reduces the cell damage caused by oxidative stress. Despite its biological effects in wound healing, resveratrol has limitations, such as low aqueous solubility, rapid degradation, and poor bioavailability [10]. To overcome these limitations, resveratrol has been incorporated into wound dressings using scaffolds, nanovesicles, and hydrogels due to their biocompatibility, biodegradability, and tissue permeability, all of which contribute to a reduced risk of infection [11]. These challenges can also be addressed using nanomaterials such as titanium dioxide nanoparticles (TiO_2_ NPs) as controlled drug release systems. TiO_2_NPs have gained interest due to their physical stability, antimicrobial properties, and non-toxic nature, which promotes cell growth [12]. A recent study by Fletes-Vargas et al. [13] produced a physical chitosan hydrogel loaded with TiO_2_ NPs containing resveratrol-rich hydroalcoholic extract from *P. cuspidatum* roots. The results indicated that the incorporation of TiO_2_ NPs loaded with resveratrol extract did not impact on the hydrogel microstructure and maintained the equilibrium water content and low hemolysis, making them suitable for wound dressing applications.

Until now, no studies have reported the biological properties and potential wound-healing applications of TiO_2_ NPs as nanocarriers for bioactive compounds in ethanolic extract from dried *P. cuspidatum* roots. In the present study, TiO_2_ NPs were loaded with a hydroalcoholic extract from dried *P. cuspidatum* roots using ultrasonication. The hemocompatibility, antioxidant activity (via DPPH and ABTS assays), and resveratrol release were evaluated. The antibacterial activities of the TiO_2_-loaded extract NPs and the *P. cuspidatum* hydroalcoholic extract were compared against commonly found Gram-positive and Gram-negative skin bacteria, and their antifungal activities were also examined.

## 2. Materials and Methods

### 2.1. Reagents

Titanium dioxide nanoparticles, DPPH (2,2-diphenyl-1-picrylhydrazyl) radical, resazurin (7-hydroxy-3H-phenoxazin-3-one-10-oxide), ABTS 2,2′-azino-bis(3-ethylbenzothiazoline)-6-sulfonic acid, and Trolox (6-hydroxy-2,5,7,8-tetramethylchroman-2-carboxylic acid) were acquired from Sigma Aldrich (Saint Louis, MO, USA). Potassium persulfate was obtained from Neon (Suzano, Brazil). A red blood cell suspension (DiaCell^®^) was acquired from Bio-Rad (Lagoa Santa, Brazil), and saponin was purchased from INLAB (São Paulo, Brazil). BHI and Muller–Hinton broth were obtained from Himedia Laboratories (Thane, India). DMEM, fetal bovine serum (FBS), and trypsin–EDTA solution were obtained from Sigma-Aldrich. All the other reagents were of analytical grade. 

### 2.2. Preparation of TiO_2_-Loaded Extract NP*s* from P. cuspidatum Hydroalcoholic Extract

The hydroalcoholic extract (60% ethanol, 40% water) from the dry roots of *P. cuspidatum* was prepared as described by Fletes-Vargas et al. [14] with slight modifications. The extract was diluted at a ratio of 11.5:1 in 60% ethanol. The TiO_2_ NPs were combined with the diluted extract at a 1:1 dry mass ratio, resulting in a final resveratrol concentration of 3.4 mg/mL. The mixture was sonicated for 30 min at 128.5 W and 45 °C using an Elmasonic p70H (37 kHz; Singen, Germany). The suspension (TiO_2_-loaded extract NPs) was centrifuged at 4000 rpm for 10 min, and the pellet was dried at 50 °C for 24 h [13].

### 2.3. Antioxidant Assay

#### 2.3.1. DPPH Radical Scavenging Activity

The radical scavenging activity (RSA) was evaluated by monitoring the discoloration of the DPPH radicals in an ethanol solution. In a 96-well plate, 200 µL of the DPPH solution (60 µmol/L) and 50 µL of each sample (including Trolox standard solutions, TiO_2_-loaded extract NPs, TiO_2_ NPs, and ethanol as a control) were added in triplicate. After 30 min of incubation in the dark at room temperature, absorbance was measured at 517 nm using a microplate reader (Hexis Científica; Jundiaí, Brazil). Trolox equivalent antioxidant capacity (TE; mg/g) was determined from a standard curve. Results were expressed as % inhibition relative to DPPH radicals, and the IC_50_ values were calculated according to Bhatnagar et al. [15].

#### 2.3.2. ABTS^+^ Radical Scavenging Activity

The ABTS analysis was performed by mixing 5 mL of ABTS^+^ solution (7 mM) with 88 µL of potassium persulfate solution (140 mM). The mixture was kept in the absence of light at room temperature for 16 h. Then, 1 mL of this solution was diluted in ethanol to an absorbance of 0.70 ± 0.05 at 734 nm. In a 96-well plate, 200 µL of the ABTS^+^ solution (7 µM) and 50 µL of each sample (TiO_2_-loaded extract NPs, TiO_2_ NPs, Trolox solutions, and ethanol as a control) were added in triplicate. After 7 min of incubation in the dark at room temperature, the absorbance was determined at 734 nm using a microplate reader (Hexis Científica; Jundiaí, Brazil). TE (mg/g) was determined from the Trolox analytical curve, and the results were expressed as % inhibition relative to ABTS^+^ radicals, and IC_50_ values were obtained [15].

### 2.4. Hemolytic Assay

The membrane toxicity of the samples was assessed according to Scalise et al. [16] with slight modifications. In a 96-well microplate, 100 μL of TiO_2_-loaded extract NPs and TiO_2_ NPs (concentrations ranging from 2.5 to 20 mg/mL) and ethanolic *P. cuspidatum* extract (concentrations ranging from 1.25 to 10 mg/mL) were added to a 3% red blood cell suspension. Saline solution (0.9% *w*/*v*) and saponin (1% *w*/*v*) served as negative and positive controls, respectively. After shaking for 3 h at 400 rpm and 25 °C, samples were centrifuged at 4000 rpm for 15 min at 18 °C. Supernatants were incubated for 30 min at room temperature for hemoglobin oxidation, and the absorbance of the released oxygenated hemoglobin (ROH) was measured at 540 nm using a UV-Vis spectrophotometer (Biospectro; Curitiba, Brazil). The hemoglobin release (%) was calculated according to Scalise et al. [16] with the following equation:

(1) $$ROH\;\left(\%\right)=\frac{{AB}_{s}-{AB}_{0}}{{AB}_{100}-{AB}_{0}}\times 100\%$$

where *AB_s_* represents the absorbance of the sample, *AB_0_* is the absorbance of the negative control (saline), and *AB_100_* is the absorbance of the positive control.

### 2.5. Resveratrol Release Assay

Resveratrol release from TiO_2_-loaded extract NPs was evaluated by measuring absorbance at 306 nm. A dispersion of TiO_2_-loaded extract NPs (100 mg/mL in ethanol) was prepared and stored at 8 °C and 37 °C. Absorbance was measured on different days (0, 2, 4, 6, and 9 days) using a microplate reader (Hexis Científica, Jundiaí, Brazil).

### 2.6. Antibacterial Assay

#### 2.6.1. Bacterial Strains

The antibacterial activity of the ethanolic *P. cuspidatum* extract, TiO_2_ extract-loaded NPs, and TiO_2_ NPs was evaluated against the Gram-negative strains *Pseudomonas aeruginosa* (ATCC 27853), *Escherichia coli* (ATCC 25922), and *Salmonella typhimurium* (ATCC 14028), and the Gram-positive strain *Staphylococcus aureus* (ATCC 29313). The strains were cultured in Mueller–Hinton broth at 37 °C.

#### 2.6.2. Microdilution Assay

The minimum inhibitory concentrations (MICs) of the ethanolic *P. cuspidatum* extract, TiO_2_ extract-loaded NPs, and TiO_2_ NPs were determined using a resazurin-based microdilution assay, since bacteria can metabolize resazurin dye in situ, indicating microbial viability [17]. The bacterial inoculum was obtained from the colonies grown on a solid BHI medium for 24 h. Then, bacteria were resuspended in sterile saline solution to a final optical density of 0.5 at 625 nm on the McFarland scale (0.08–0.1 at 625 nm). The bacterial suspension was adjusted to 1 × 10^7^ CFU/mL using Mueller–Hinton broth medium. In a 96-well microplate, 50 μL of the bacterial suspension and the samples at various concentrations were evaluated. The ethanolic *P. cuspidatum* extract was tested at 0.31–10 mg/mL; meanwhile, the TiO_2_ extract-loaded NPs and TiO_2_ NPs were tested at 3.12–100 mg/mL. Chloramphenicol (30 µg/mL) was used as positive control. The microplates were incubated for 24 h at 37 °C, and later, 30 μL of resazurin solution (0.01%) was added to each well and incubated for 24 h at 37 °C. Blue coloration indicated antibacterial activity, while pink coloration showed viable microorganisms.

#### 2.6.3. Antifungal Assay

The antifungal activity was assessed using the disk diffusion method against *Candida albicans* and *Candida glabrata*. The yeast strains were cultured on Mueller–Hinton agar at 35–37 °C in a Fanem incubator (Guarulhos, Brazil) until visible colonies formed. These colonies were suspended in sterile saline solution and inoculated onto Mueller–Hinton agar plates. Discs impregnated with the *P. cuspidatum* hydroalcoholic extract, the TiO_2_-loaded extract NPs, and the TiO_2_ NPs were placed on the plates. Amphotericin B and an unimpregnated sterile disc were used as positive and negative controls, respectively. Inhibition zones were measured (mm) using a caliper after 24 h of incubation at 35–37 °C.

### 2.7. In Vitro Cytotoxic Assay

The effects of TiO_2_-loaded extract NPs and bulk TiO_2_ NPs on the viability of cells were assessed using a resazurin reduction assay [18]. The treatments were diluted using DMEM to obtain different concentrations. The NCTC clone 929 fibroblast cell line (L-cell, L929, CCL-1, ATCC), derived from connective mouse tissue, was used for toxicity testing. The L-929 cell line was cultured in DMEM supplemented with 10% FBS and 1% penicillin/streptomycin. The cells were incubated until they reached 80% confluency and seeded in a 96-well plate at 5 × 10^3^ cells per well at 37 °C and 5% CO_2_. After 24 h of incubation, DMEM was replaced with 100 µL of the samples at different concentrations and incubated for 24 h under the previous conditions. Later, 20 µL of resazurin solution (0.2 mg/mL) was added and incubated for 3 h at 37 °C. The fluorescence of the metabolized resazurin was measured (excitation at 520 nm and emission at 590 nm). DMEM medium and DMSO (10%) were used as negative and positive controls, respectively. The cell viability (%) was calculated as follows:

(2) $$\%\;\mathrm{cell}\;\mathrm{viability}=\frac{I_{s}}{I_{c}}\times 100\%$$

where *I_s_* is the absorbance of the cells exposed to nanoparticles and *I_c_* is the absorbance of the cells without exposure to the treatments.

### 2.8. Statistical Analysis

All the experiments were performed in triplicate, and the results are expressed as the means ± standard deviations. Statistical differences were determined by ANOVA followed by Tukey’s test. The data were analyzed using Origin Pro 2018 software (Origin Lab Corporation, Northampton, MA, USA), and the differences among the means were evaluated (*p* ≤ 0.05).

## 3. Results and Discussion

### 3.1. Antioxidant Activity

Polyphenols, such as resveratrol, are plant-derived antioxidants that act as electron donors, efficiently inactivating free radicals, reducing oxidative stress, and preventing molecular oxidation [19]. An increase in ROS induces oxidative stress, which can damage biomolecules such as proteins and DNA, contributing to chronic and neurodegenerative diseases [20]. Moreover, elevated oxidative stress is associated with delayed wound healing [21]. The antioxidant activity of the optimized *P. cuspidatum* extract was previously reported by our research group, and demonstrated high radical scavenging activity: 135.1 μg TE/mL (IC_50_ = 78 μg TE/mL) for DPPH, and 230.4 μg TE/mL (IC_50_ = 158 µg TE/mL) for ABTS^+^ [14].

**Figure 1:**
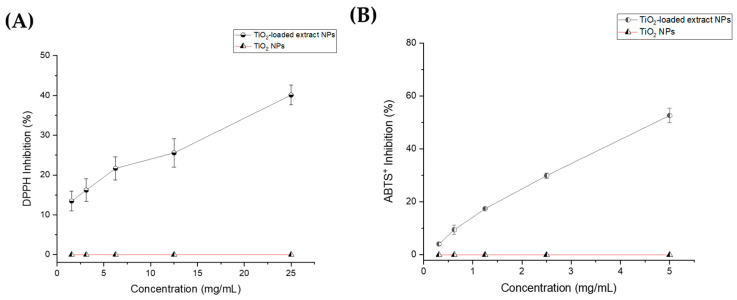
Radical antioxidant activity. (**A**) DPPH antioxidant assay for TiO_2_-loaded extract NPs and TiO_2_ NPs, and (**B**) ABTS^+^ radical assay for TiO_2_-loaded extract NPs and TiO_2_ NPs. All data represent means ± SEM (n = 6).

The results of the DPPH inhibition activity for the TiO_2_-loaded extract NPs, TiO_2_ NPs, and Trolox standard are shown in Figure 1A. TiO_2_-loaded extract NPs displayed a dose-dependent response, with radical scavenging inhibition ranging from 13.45 ± 2.48 to 40.6 ± 2.54% at concentrations from 1.56 to 25 mg/mL (R^2^ = 0.9930). The calculated IC_50_ for TiO_2_-loaded extract NPs was 62.31 mg /mL, indicating a significant antioxidant activity. In contrast, bulk TiO_2_ NPs did not show DPPH inhibition activity at the evaluated concentrations. Similarly, the results of the ABTS^+^ radical assay indicate higher antioxidant activity in a dose-dependent manner, ranging from 4.05 ± 0.58 to 52.62 ± 2.76% at concentrations from 0.3125 to 5.0 mg/mL. Also, the IC_50_ value for TiO_2_-loaded extract NPs was 4.8 mg/mL (R^2^ = 0.9963). In contrast, no antioxidant effect was observed for the bulk TiO_2_ NPs. Trolox calibration curves for DPPH and ABTS assays were evaluated, obtaining R² values of 0.9958 and 0.9980, respectively.

The TiO_2_-loaded extract NPs exhibited significant differences in IC_50_ values between the two methods used (DPPH and ABTS^+^ assays). The IC_50_ obtained from the DPPH assay was considerably lower than that from the ABTS^+^ assay. This disparity can be attributed to the higher solubility of bioactive compounds, such as resveratrol and piceid, from TiO_2_-loaded extract NPs in ethanol, which was used as the solvent. The interaction of these bioactive compounds with the ABTS^+^ cation radical may be limited due to their lower solubility in aqueous media [22].

### 3.2. Hemolytic Actiivit

Nanoparticles that reach the bloodstream can interact with blood cells, particularly erythrocytes, potentially leading to biochemical, morphological, and physiological disruptions [23]. Hemolytic activity induced by nanoparticles is considered a critical factor for evaluating the hemocompatibility of nanomaterials for biotechnology applications [24]. In this study, red blood cell lysis was assessed upon contact with the different samples, serving as an indicator of membrane toxicity and hemoglobin leakage. Table 1 presents the results of the hemolytic activity and the hemoglobin release (ROH %) for the *P. cuspidatum* extract, TiO_2_-loaded extract NPs, and TiO_2_ NPs.

The results show that the *P. cuspidatum* extract exhibited a dose-dependent hemolytic effect, with values ranging from 2.3 ± 0.7 to 42.3 ± 4.3% between 1.25 and 10 mg/mL of resveratrol, while TiO_2_-loaded extract NPs displayed hemolysis values ranging from 1.3 ± 0.2 to 3.9 ± 0.6% between 2.5 and 20 mg/mL of resveratrol. Bulk TiO_2_ NPs showed hemolysis values of 15.6 ± 4.6% at 20 mg/mL and decreased to 4.6 ± 0.8% at the lowest concentration (2.5 mg/mL). In this sense, Ghosh et al. [25] evaluated the hemolytic effect of TiO_2_ NPs on human erythrocytes. They reported that cell lysis occurs through spherocytosis and echinocytosis. Additionally, an interaction between TiO_2_ NPs and hemoglobin was observed, which caused hemolysis by cell membrane damage and impaired cell oxygen transport.

Our results suggested that the incorporation of *P. cuspidatum* extract into TiO_2_ nanoparticles significantly reduces membrane toxicity, potentially enhancing their safety for biomedical applications. Furthermore, the data indicate that TiO_2_-loaded extract NPs exhibited good hemocompatibility, supporting their potential use in wound healing therapies.

### 3.3. Resveratrol Release

**Figure 2:**
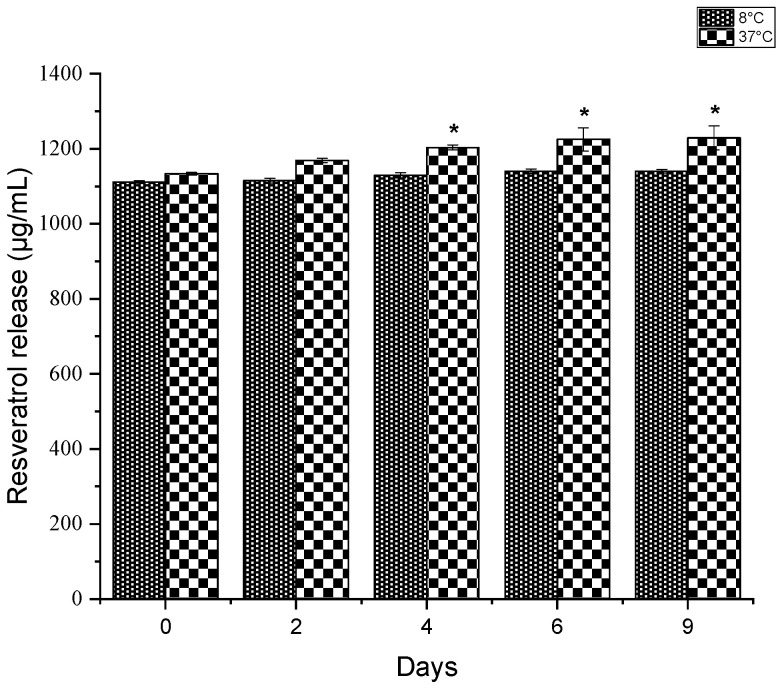
Resveratrol release from TiO_2_-loaded extract NPs over time. The values represent means ± SEM. * Statistically significant (*p* ≤ 0.05).

Despite its biological benefits, resveratrol is rapidly metabolized (about 8–14 min) following oral intake, exhibits a short half-life, and low aqueous solubility [26]. To address these limitations, topical delivery presents an effective alternative for achieving controlled resveratrol release. In this study, resveratrol release from TiO_2_-loaded extract NPs was monitored over 9 days at 8 °C and 37 °C. Figure 2 shows the gradual resveratrol release from TiO_2_-loaded extract NPs. The results revealed a sustained release profile at 37 °C, with notable increases in resveratrol release on days 4, 6, and 9. In contrast, no statistically significant differences in release were observed at 8 °C (*p* ≤ 0.05).

This suggests that high temperatures increase the release rate of resveratrol from the nanoparticles, which could enhance its effectiveness in therapeutic applications at physiological temperatures. Furthermore, Soldati et al. [27] encapsulated resveratrol extract from *Theobroma grandiflorum* using solid lipid nanoparticles as nanocarriers. The in vitro results demonstrated a controlled release over 24 h, with an initial burst phase associated with the hydroxyl groups promoting localization near the hydrophilic surface.

### 3.4. Antibacterial Activity

The skin microbiome and its microenvironment can negatively affect the wound healing process as skin damage facilitates bacterial proliferation and colonization, leading to infection, inflammation, and delayed wound closure [28]. *S. aureus* and *S. typhimurium* can be found in the skin microbiota; however, bacterial overgrowth is associated with chronic wound infections and pro-inflammatory responses that damage skin tissue. Meanwhile, *P. aeruginosa* and *E. coli* are frequently found in pathogenic burn wounds and contribute to impaired skin barrier recovery [29]. Although antibiotics are commonly used to treat infections and inflammation in wounds, their prolonged use is associated with bacterial resistance and adverse effects. Plant-derived extracts may offer a promising alternative, acting as immune response modulators without promoting resistance [30]. In this context, resveratrol has demonstrated potential antibacterial activity, including the inhibition of pathogenic bacteria such as *S. aureus*, a known causative agent of pneumonia [7,31].

In this study, the antibacterial activities of *P. cuspidatum* extract, TiO_2_-loaded extract NPs, and TiO_2_ NPs were evaluated against *P. aeruginosa*, *E. coli*, *S. aureus*, and *S. typhimurium*. Table 2 summarizes the MICs of the *P. cuspidatum* extract, the TiO_2_-loaded extract NPs, and the TiO_2_ NPs against the tested bacterial strains. The results show that the *P. cuspidatum* extract exhibited a high antibacterial activity, with MIC values of 5 mg/mL against all tested strains. The MIC obtained for the TiO_2_-loaded extract NPs was 50 mg/mL against *P. aeruginosa*, *E. coli*, *S. aureus*, and *S. typhimurium*. In contrast, no antibacterial activity was observed for TiO_2_ NPs at the same concentrations. The antibacterial effect of the TiO_2_-loaded extract NPs is likely associated with the synergistic effect of the bioactive compounds in the *P. cuspidatum* root extract, including resveratrol and piceid.

A study by Shan et al. [32] indicated that the crude extract of *P. cuspidatum* roots exhibited excellent antibacterial activity against both Gram-positive (*B. cereus*, *L. monocytogenes*, and *S. aureus*) and Gram-negative bacteria (*E. coli* and *S. anatum*), which was compared with the activity observed with resveratrol, piceid, emodin, and physcion standards. The authors suggested that the antibacterial activity of the *P. cuspidatum* extract is associated with the major bioactive compounds identified in the crude extract. Similarly, Su et al. [33] evaluated the antimicrobial activity of a *P. cuspidatum* extract against drug-resistant nosocomial bacteria and reported a broad antimicrobial spectrum against all tested bacterial strains at concentrations ranging from 0.1 to 3.5 mg/mL, without inducing resistance to its bioactive compounds. Üstündağ et al. [34] evaluated the antioxidant and anti-inflammatory activities of silver nanoparticle-enhanced resveratrol (AgNP-RV) using a sepsis-induced lung injury model in animals. Their findings showed that AgNP-RV alleviated sepsis-induced oxidative stress and reduced tissue damage more effectively than resveratrol alone.

### 3.5. Antifungal Activity

**Figure 3:**
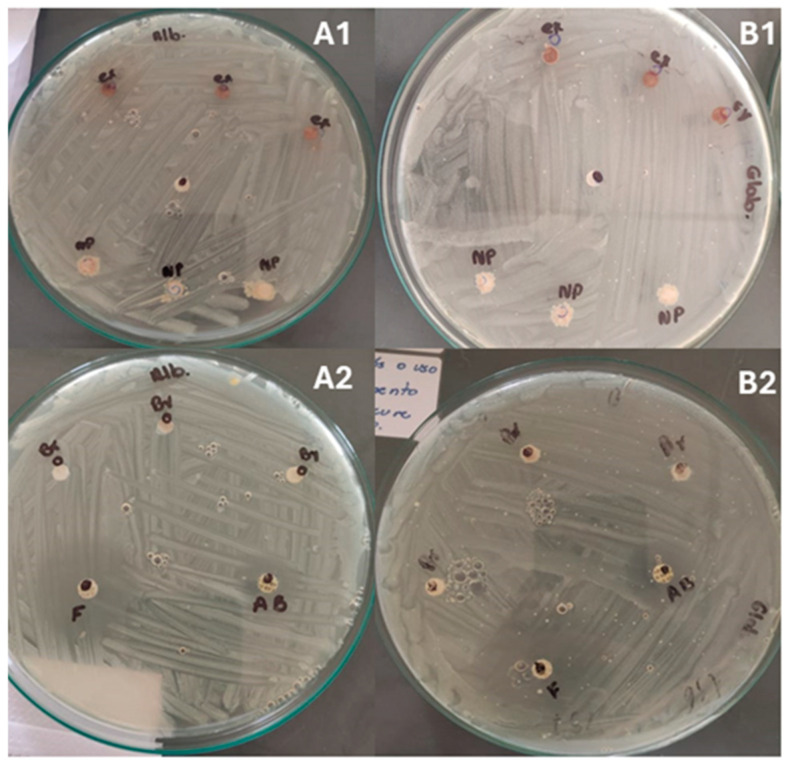
Antifungal effect of the *P. cuspidatum* extract, TiO_2_ NPs, and TiO_2_-loaded extract NPs against *C. albicans* and *C. glabrata*. (**A1**,**A2**): fungicidal effect on *C. albicans;* (**B1**,**B2**): antifungal effects on *C. glabrata*.

Fungi are a natural component of a healthy skin microbiome, where recent studies suggested that fungi may delay the healing of chronic wounds by protecting and facilitating the attachment of pathogenic bacteria. For example, *Candida* spp., particularly opportunistic pathogens such as *C. albicans* and *C. glabrata*, are commonly found in diabetic wounds, and their presence has been associated with secondary bacterial infections due to compromised skin barriers [35]. Wounds infected with *C. albicans* and *C. glabrata* exhibit a slower healing rate compared to those infected with bacteria such as *P. aeruginosa* or *S. aureus.* Shevelev et al. [36] reported that treatment with resveratrol can promote wound closure in fungal-infected wounds, approaching the healing efficiency observed in uninfected wounds. In the present study, the antifungal activities of *P. cuspidatum* extract, TiO_2_-loaded extract NPs, and bulk TiO_2_ NPs were evaluated against *C. albicans* and C. glabrata. The results indicated that none of the samples demonstrated fungicidal activity against *C. albicans* and *C. glabrata* (Figure 3).

In contrast, Lee and Kim [37] demonstrated that *P. cuspidatum* extract inhibit *C. albicans* adhesion and biofilm formation, inducing cell membrane damage. On the other hand, Collado-González et al. [38] reported that resveratrol lacks antifungal activity against *C. albicans* CAI.4 strain at concentrations ranging from 10 to 40 µg/mL, although antifungal effects were observed at 400 µg/mL in comparison with the control amphotericin B at 5 µg/mL. These findings suggest that the variability across studies may be attributed to the complex composition of *P. cuspidatum* root extract, which includes resveratrol, piceid, emodin, and other derivatives, and the synergistic interactions of these bioactive compounds.

### 3.6. Cytotoxicity Assay

When the skin is damaged, immune cells recruit fibroblasts to synthesize the extracellular matrix, thereby promoting tissue repair. In addition to their role in wound healing, fibroblasts are essential for maintaining skin homeostasis [39]. To evaluate the biocompatibility of TiO_2_-loaded extract NPs and TiO_2_ NPs, fibroblasts were used as model cells due to their key role in the wound healing process.

**Figure 4:**
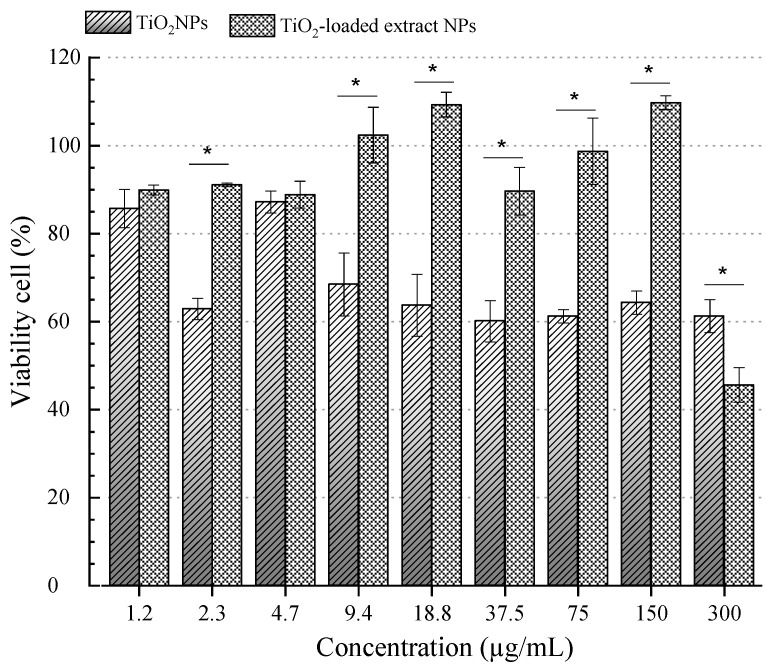
Viability of cells treated with TiO_2_-loaded extract NPs and TiO_2_ NPs at different concentrations against the L-929 cell line for 24 h. * Statistically significant (*p* ≤ 0.05).

In this study, the cytotoxic effects of TiO_2_-loaded extract NPs and TiO_2_ NPs were evaluated in a dose-dependent manner (Figure 4). The viability of cells treated with TiO_2_ NPs was below 70% from 9.4 to 300 µg/mL, indicating cytotoxicity according to the ISO 10993-5:2009 guidelines [40].

In contrast, fibroblast viability remained above 70% for TiO_2_-loaded extract NPs at concentrations ranging from 1.5 to 150 µg/mL, with a significant decrease only observed at 300 µg/mL (*p* ≤ 0.05). Similarly, Jin et al. [41] evaluated the cytotoxic impact of TiO_2_ NPs on L-929 mouse fibroblast cells. The authors reported morphological modifications in treated cells, such as shrinkage, lysosome increase, and fragmented chromatin-associated necrosis cell death mechanism. Additionally, oxidative stress and ROS levels increased significantly at concentrations above 60 µg/mL of TiO_2_ NPs, while antioxidant enzyme levels decreased. Similarly, Chen et al. [42] demonstrated that TiO_2_ NPs induce DNA damage via oxidative mechanisms. However, resveratrol molecules canmitigate this damage. Giovannelli et al. [43] reported that resveratrol protects human fibroblasts from DNA oxidative stress by reducing histone and p53 protein acetylation. Additionally, 3T3 fibroblast cells treated with resveratrol showed enhanced proliferation and migration rates, accompanied by decreased oxidative stress, suggesting their potential application in wound healing [44].

## 4. Conclusions

Incorporating *P. cuspidatum* root extract into TiO_2_ nanoparticles significantly enhances their antioxidant and hemocompatibility properties compared to bulk TiO_2_ nanoparticles. Moreover, the *P. cuspidatum* extract alone exhibited excellent antibacterial properties against the tested strains, compared to TiO_2_-loaded extract NPs. Additionally, antifungal activity against *C. albicans* and *C. glabrata* was not observed. The temperature-dependent release of resveratrol suggested an additional advantage for therapeutic applications at body temperature. These findings highlight the potential of *P. cuspidatum* root extract and TiO_2_-loaded extract NPs for future applications in wound management, including acne treatment, diabetic ulcers, and burn care. Furthermore, TiO_2_-loaded extract NPs can be loaded in biocompatible hydrogels, ointments, or fibers, especially where antioxidant, antimicrobial, and biocompatible properties are required.

## Figures and Tables

**Table 1:** Percentage of hemoglobin released (ROH%) after contact with the *P. cuspidatum* extract, the TiO_2_-loaded extract NPs, and the TiO_2_ NPs.

*P. cuspidatum* Extract		TiO_2_-Loaded Extract NPs		TiO_2_ NPs	
(Resveratrol mg/mL)	ROH (%)	(mg/mL)	ROH (%)	(mg/mL)	ROH (%)
10	42.3 ± 4.3 ^a^	20	3.9 ± 0.6 ^a^	20	15.6 ± 4.6 ^a^
5	24.8 ± 1.1 ^b^	10	3.2 ± 1.3 ^a^	10	7.96 ± 1.3 ^b^
2.5	7.5 ± 1.2 ^c^	5	2.0 ± 0.6 ^a^	5	5.9 ± 0.9 ^b^
1.25	2.3 ± 0.7 ^d^	2.5	1.3 ± 0.2 ^a^	2.5	4.6 ± 0.8 ^b^

The values represent the means ± S.E.M. (n = 3). ^a,b,c,d^ The same letters are considered not significantly different according to the Tukey HSD test (*p* < 0.05).

**Table 2:** MICs of the tested strains after contact with the *P. cuspidatum* ethanolic extract, TiO_2_-loaded extract NPs, and TiO_2_ NPs.

Bacterial Strain	*P. cuspidatum* Extract (Resveratrol mg/mL)	TiO_2_-Loaded Extract NPs (mg/mL)	TiO_2_ NPs (mg/mL)
*P. aeruginosa*	5	50	ND
*E. coli*	5	50	ND
*S*. *aureus*	5	50	ND
*S*. *typhimurium*	5	50	ND

ND = no activity detected.

## Data Availability

Data will be made available on request.
